# In Search for the Retrievable Memory Trace in an Insect Brain

**DOI:** 10.3389/fnsys.2022.876376

**Published:** 2022-06-08

**Authors:** Randolf Menzel

**Affiliations:** Institute Biology – Neurobiology, Freie Universität Berlin, Berlin, Germany

**Keywords:** olfaction, reward neuron, honeybee, mushroom body, Ca imaging, synapses, identified neurons, patterns of change

## Abstract

The search strategy for the memory trace and its semantics is exemplified for the case of olfactory learning in the honeybee brain. The logic of associative learning is used to guide the experimental approach into the brain by identifying the anatomical and functional convergence sites of the conditioned stimulus and unconditioned stimulus pathways. Two of the several convergence sites are examined in detail, the antennal lobe as the first-order sensory coding area, and the input region of the mushroom body as a higher order integration center. The memory trace is identified as the pattern of associative changes on the level of synapses. The synapses are recruited, drop out, and change the transmission properties for both specifically associated stimulus and the non-associated stimulus. Several rules extracted from behavioral studies are found to be mirrored in the patterns of synaptic change. The strengths and the weaknesses of the honeybee as a model for the search for the memory trace are addressed in a comparison with *Drosophila*. The question is discussed whether the memory trace exists as a hidden pattern of change if it is not retrieved and whether an external reading of the content of the memory trace may ever be possible. Doubts are raised on the basis that the retrieval circuits are part of the memory trace. The concept of a memory trace existing beyond retrieval is defended by referring to two well-documented processes also in the honeybee, memory consolidation during sleep, and transfer of memory across brain areas.

## Introduction

Neuroscientists searching for the memory trace have been particularly strongly attracted by the associative forms of learning. This makes sense because in associative learning, two stimuli converge in time, and since these stimuli are encoded in separate neural pathways, the respective neural activities have to converge in time and in brain space to lead to lasting neural changes, the memory trace (Hebb, 1949; Kandel, 1976). Such an experimental strategy does not exclude the possibility of other neural mechanisms than associativity in other forms of learning like sensory preexposure, exploratory learning, procedural learning, rule learning or declarative learning, and these forms of learning will establish their particular memory traces (Squire, 1986; Gallistel, 1990; Anderson, 2013). Whether these memory traces comprise neural mechanisms based on associative mechanisms is an open question and worth exploring.

Localizing the memory trace is an important step in a functional analysis, and the recent developments in knocking-out, reactivating, recording, and stimulating selected neurons in identified networks involved in acquisition, memory formation and retrieval lead to a great step in current cognitive neuroscience (Fiala, 2007; Garner et al., 2012; Aso and Rubin, 2016). Focusing the search for the memory trace on associative forms of learning offers opportunities about the potential locations of the memory trace, namely, at the anatomical convergence sites of the pathways for the stimuli involved, the to-be-learnt stimulus (conditioned stimulus, CS) and the evaluating stimulus (unconditioned stimulus, US). The complete knowledge of the pathways involved in associative learning of olfactory stimuli make the fruit fly Drosophila to an excellent model for such studies on the level of identified neurons (Aso et al., 2014b; Aso and Rubin, 2016; Gerber and Aso, 2017) and the synapses between the sensory and evaluating neurons (Barth et al., 2014; Pech et al., 2015).

The anatomical and function inter-cellular convergence sites in the brain are comprised by synapses. Coding any sensory stimulus or motor pattern requires the activation of subsets of neurons *via* their subsets of synaptic contacts. The memory store may well include hierarchies of intracellular molecular pathways, but their contributions do not act on sensory or motor coding other than *via* the transfer to the inter-cellular space by synapses. Ultimately, a memory trace will be implemented in a pattern of multiple synapses that are built *de novo*, that become non-functional and/or that are up or downregulated in the consequence of learning. The semantics of the memory trace should, therefore, be reflected in the pattern of synaptic change during learning.

I shall follow this argument taking advantage of an insect brain, that of the honeybee. Insect brains are small, many neurons can be identified even at the level of the single neuron. Their connectivity pattern is often well-known, and synaptic contacts are known to some extent. The brain of the fruit fly *Drosophila* is an exceptional case since practically all neurons and their synapses have been captured on the sub-microscopical level (Zheng et al., 2018). The brain of the honeybee, the subject here, is far from reaching such a level of knowledge, but still many neurons are well-described and some of them individually identified (Brandt et al., 2005; Rybak et al., 2010, https://insectbraindb.org/app/). Thus, the neuroanatomy on the cellular and circuit level will guide us in our search for the memory trace in the honeybee brain. Honeybees are attractive for these studies because of their rich and highly flexible behavior that is accessible under fully natural and more restricted laboratory conditions. Reward learning is not limited to the level of satiation of the individual animal because under natural test conditions they collect food (nectar and pollen) for the colony which makes them continuously motivated for food sampling and learning. Multiple forms of learning are well-documented including rule learning, exploratory learning, and learning in the social context (Menzel and Giurfa, 2001; Giurfa, 2015).

## Associative Learning in Bees

**Figure 1:**
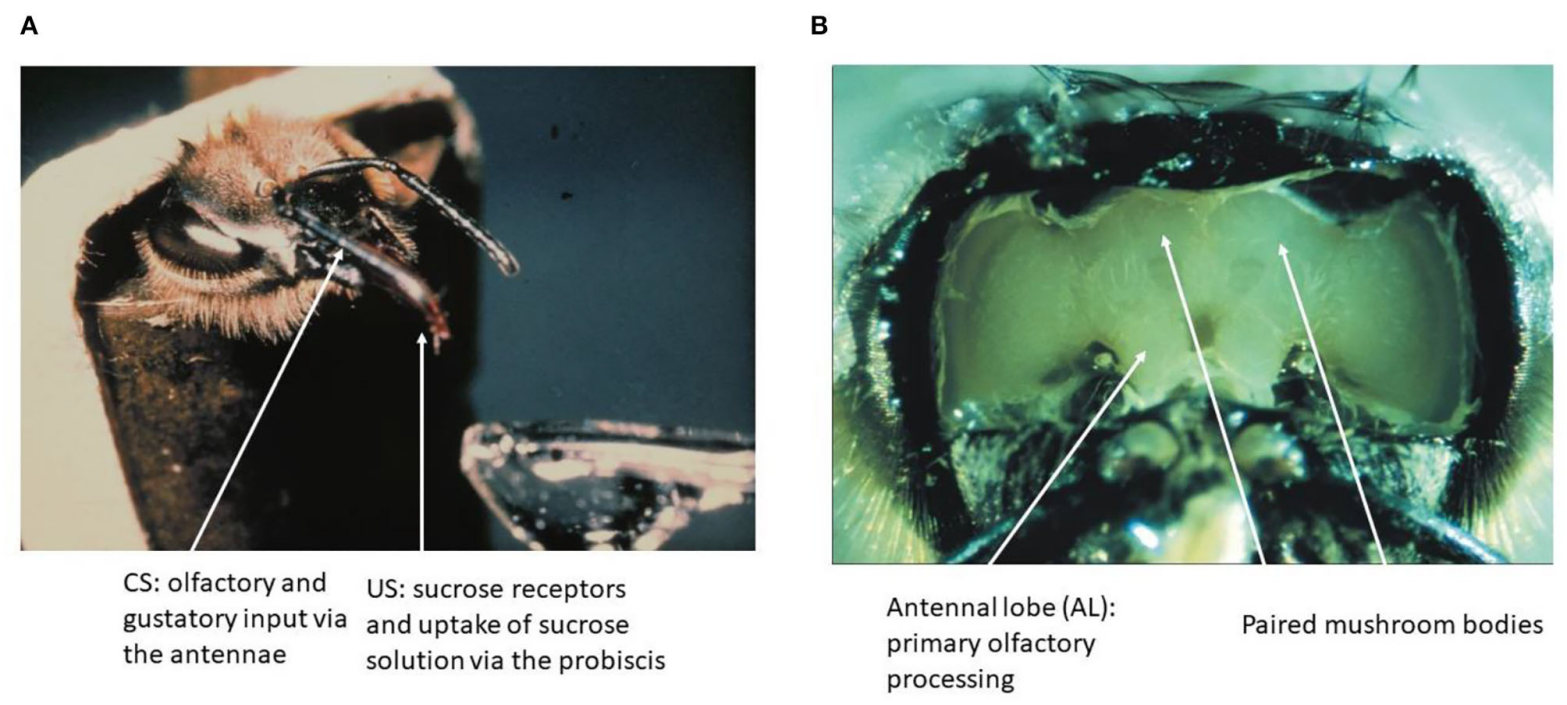
The olfactory conditioning and the honeybee brain. **(A)** A hungry bee fixed to a tube extends its proboscis (tongue) when the sucrose receptors on the antennae are stimulated. Forward pairing of an odor puff (as CS) with sucrose solution (as US) leads to fast learning as expressed by exposing the bee to the CS alone later. **(B)** The brain of the bee can be exposed to neurophysiological recordings without compromising the learning of an odor stimulus.

Learning in the honeybee is embedded in the natural context of flower pollination. The general pollinators such as the honeybee and all other flying hymenopteran insects select flowers by searching for them and learning their floral cues (odor, color, visual pattern, construction of the flower, and surface of their petals) if they are rewarded by nectar or/and pollen (Faegri and van der Pijl, 1978; Feinsinger, 1983; Kevan and Menzel, 2012). Odor is a particular salient cue that is often learned by a single pairing with reward (von Frisch, 1919). Honeybees learn and discriminate a seemingly unlimited number of odors (natural and artificial), categorize odor mixtures as unique stimuli, identify odors within 250 ms and odor sequence within 6 ms (Galizia and Menzel, 2000). Associative odor learning can be moved to the laboratory by harnessing bees to tubes, starving them from food overnight and expose them to a puff of odor shortly before stimulating their antennae with sucrose solution and then feeding them *via* their tongue (proboscis) (Bitterman et al., 1983) (Figure 1A).

The antennae contain a large number (~60,000) of odor receptors on each antenna. Sucrose receptors are located also on the antennae and on the proboscis. The hungry bees will reliably rely on a reflex of stretching the proboscis when the antennae are stimulated with sucrose solution. Bees naive for a particular odor will not extend the proboscis for the odor stimulus. Thus, the odor conditioning is a typical form of fast associative learning with the odor being the CS, and the sucrose rewards the US. Multiple associative paradigms have been applied to bees in the tube and the true nature of associativity is well-documented (Menzel, 1993; Menzel and Giurfa, 2006). I will add here just one example to highlight the complexity of odor learning under these conditions. Bees solve a bi-conditional discrimination task in which four different odors are used (A, B, C, and D). For example, when AB+, CD+ are rewarded and AC–, BD– are not rewarded, the bees solve the task (Chandra and Smith, 1998). This capacity demonstrates that the olfactory compounds were learned as the entities different from the simple sum of their elements. Similarly, a negative patterning task (A+, B+, AB–) (Deisig et al., 2003) is also learned. Such forms of associative learning can only be explained if the compound AB is treated as being different from the simple sum of its elements. Two theories were put forward to explain such data, the configural theory, which proposes that a mixture constitutes an entity different from its components (AB = X ≠ A + B) (Pearce, 1994) and the unique-cue theory, which proposes that a mixture is processed as the linear sum of its components plus a stimulus (u) that is unique to the joint presentation of the elements in the mixture (AB = A + B + u) (Whitlow and Wagner, 1972). In the case of honeybee olfactory learning, the computer simulations and experiments such as negative patterning and its variants showed that the olfactory compound learning is consistent with the unique cue theory.

## The Brain of Bees

The brain of the honeybee has a volume of about 1 mm^3^ and contains ~1 M of neurons. Many of these neurons have been characterized fully in their anatomy, and some of these neurons are known as unique identified neurons. A digital atlas of the bee brain has been established as a most versatile tool for functional analyses (Rybak et al., 2010; https://insectbraindb.org/app/). In the context of the olfactory learning and the search for the corresponding memory trace, two brain regions are of particular importance, the antennal lobe (AL) as the first-order processing area of odors and the mushroom bodies (MB) as the higher order multisensory integration area (Figure 1B). The surface of the brain can be exposed by cutting the cuticula between the dorsal ocelli, the inner rim of the compound eyes and the base of the antennae, and then removing the air sacks on top of the brain. Bees can be trained to recognize odors under these conditions, and will express their unconditioned and conditioned responses (proboscis extension response, PER). This preparation also allows for electrophysiological and opto–physiological recordings from identified neurons and neuropil areas during training and testing.

The MB have been related to insect intelligence since their discovery in the 19th century. Kenyon (1896) who provided the first detailed microscopic analyses wrote as follows: “Ever since Dujardin (1850) discovered the MB and pointed out the relation between their size and the development of insect intelligence, nearly every writer on the subject of the hexapod brain who has referred to the matter of intelligence has recognized the fact.” The additional data along these arguments came from von Alten (1910) and Howse (1975). The MB was found to be involved in the transition from short-term to an early form of long-term memory by restricting cold pulses to the MB. Retrograde amnesia was induced if cooling was applied within the first few minutes after a single trial learning (Menzel et al., 1974). It was also observed that parts of the MB increased in volume with age and experience (Durst et al., 1994; Fahrbach et al., 1995). These anatomical adaptations house in the neuropil because no neuroblasts survive adult emergence (Ganeshina et al., 2000). Interestingly, Hourcade et al. (2010) established a close relationship between synaptic structures in the olfactory input part of the MB, the lip region, and consolidation of olfactory memory in the honeybee.

## Circuit Building

### The CS-Pathway

The AL of an insect is the functional analog of the olfactory bulb in mammals. The first-level synaptic interaction of large numbers of multiple-type olfactory receptor neurons with AL interneurons serves the function of reliably coding a vast range of odorants and their mixtures, and the separation between odor identity and odor concentration. Therefore, the AL is the first-order neuropil serving basic functions of odor discrimination, categorization, generalization, and possibly learning. The synaptic contacts between the receptor axon terminals, the local interneurons and the second-order projection neurons (PNs) are organized in 163 glomeruli similar to the neural organization in the mammalian olfactory bulb (Shepherd and Grillner, 2018, Chapter 28). The output from these glomeruli comprises several tracts of PNs. We shall deal here with PNs that project *via* two tracts [the lateral antennal lobe (l-ALT) and the median antennal lobe tract (m-ALT)] to the MB and then to a lateral region of the brain, the lateral horn (LH). Each of the l- and m-ALT carry about 400 PN axons (Abel et al., 2001; Rybak, 2012; Zwaka et al., 2016, Figure 2). The major projections of l- and m-ALTs terminate in presynaptic boutons in the lip region of the MB (Figure 4C). Imaging the Ca^2+^ activity upon odor stimulation indicates a spatial and combinatorial olfactory code at the level of the glomeruli (Joerges et al., 1997; Galizia and Menzel, 2000) and thus to the PNs (Abel et al., 2001; Müller et al., 2002; Krofczik et al., 2008). Thus, the odor identity code reaches the input to the MB *via* multiple PNs in combinatorial patterns of neural activity. The PN-specific differences of the arborizations in the MB lip argues for related functional differences in addressing subsets of Kenyon cells (KCs). This appears to be different from the wiring of PNs in the Drosophila MB which keeps the spatial separation established in the glomeruli of the AL (Wong et al., 2002).

**Figure 2:**
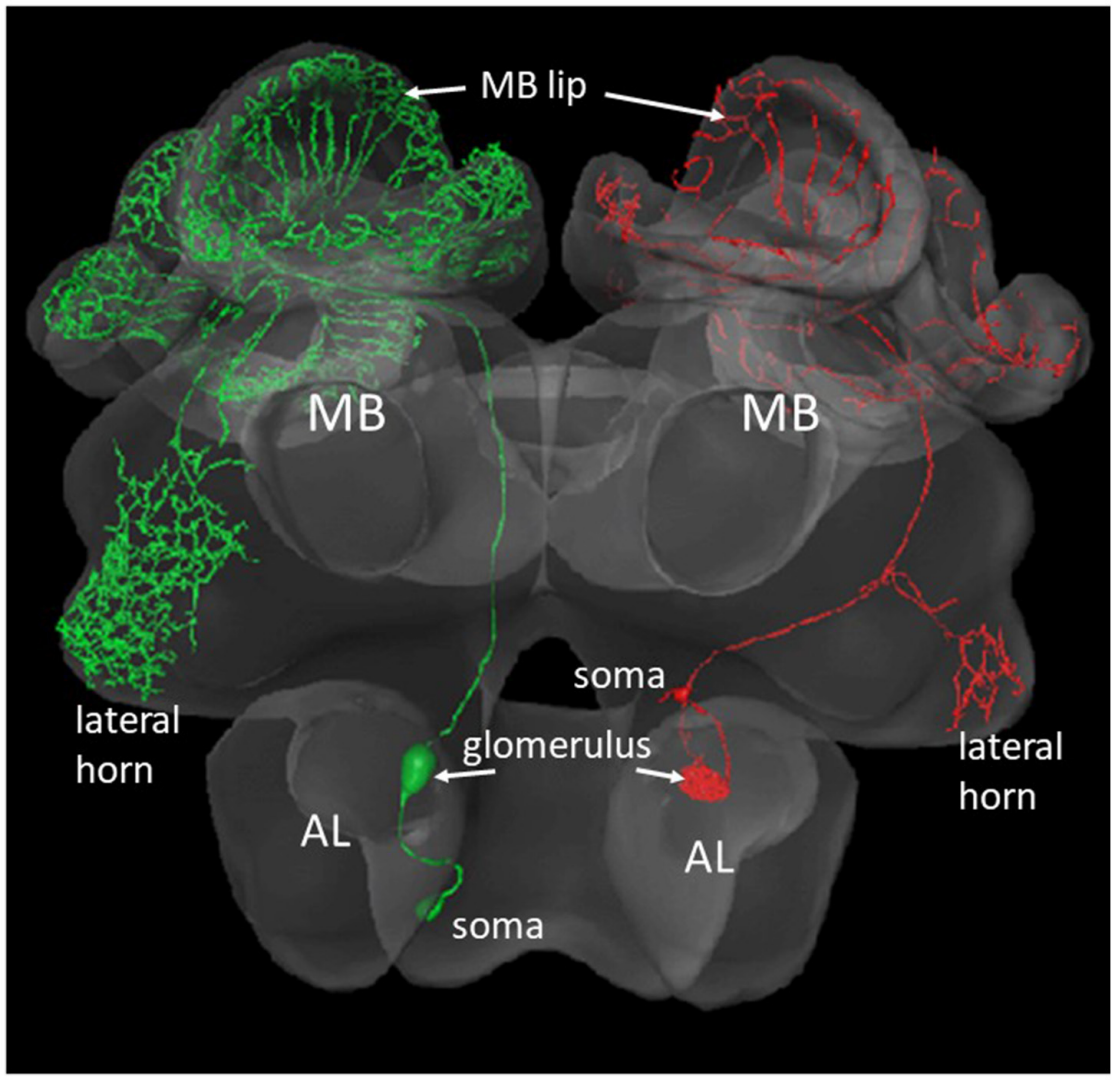
Two types of olfactory PNs that project from the AL to the MB and the lateral horn. The green neuron at the left comprises a m-ALT, the red neuron to the right a l-ALT. The blebs inside the AL show the postsynaptic arborizations of the respective neuron in a glomerulus. The small bleb at the rim of the AL shows the respective somata (Zwaka et al., 2016), https://insectbraindb.org/app/.

### The US-Pathway

**Figure 3:**
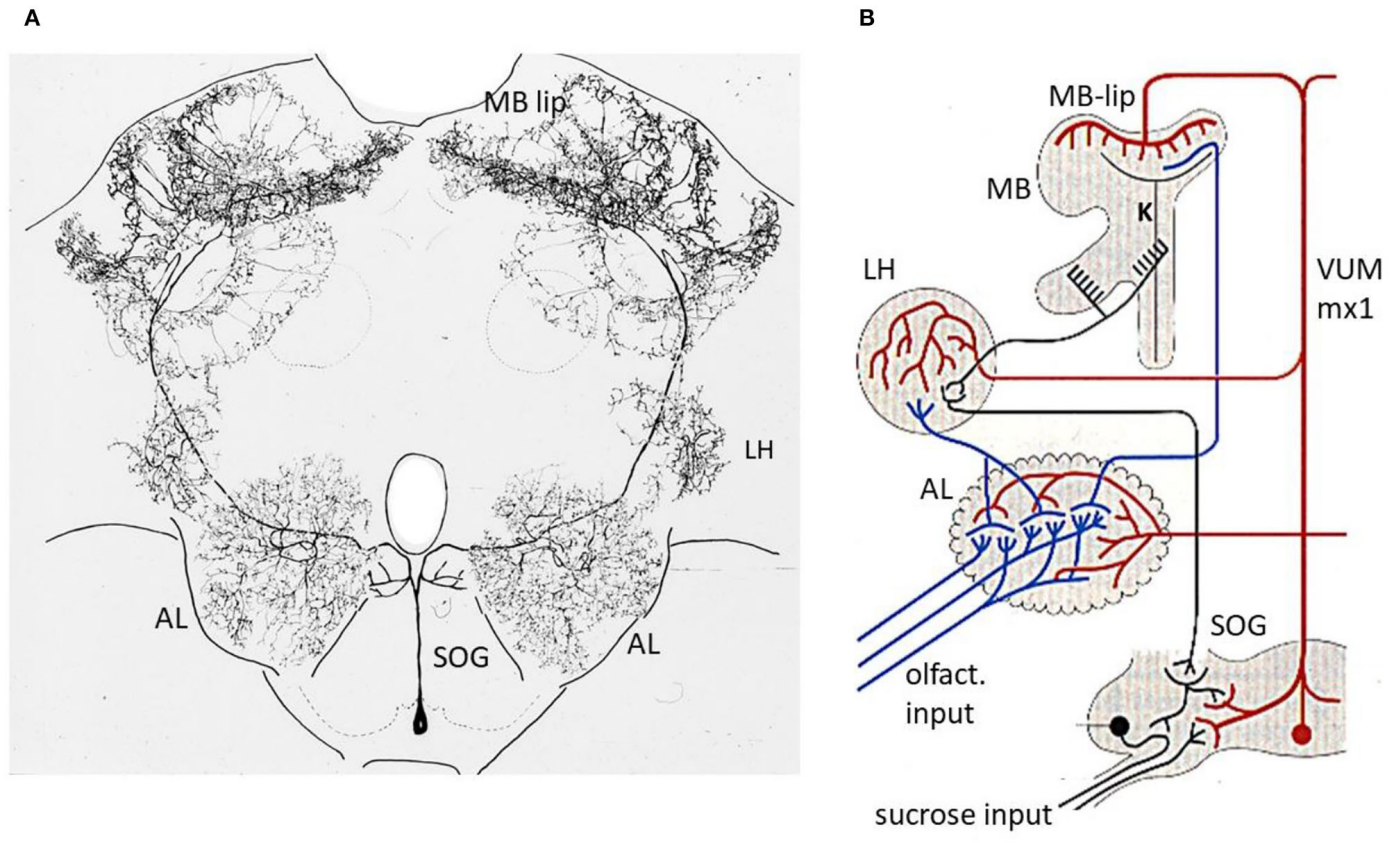
Reward pathway. **(A)** Intracellular marking of the VUMmx1 neuron (Hammer, 1993). **(B)** Convergence sites between the olfactory and reward pathways. Notice that the pathway via the MB lies in a parallel path of the higher order olfactory neurons because a subset of olfactory neurons project directly from the AL to the LH. The latter comprises one of the neuropils where descending premotor neurons originate. AL, antennal lobe; MB, mushroom body; LH, lateral horn; SOG, subesophageal ganglion. Figure 3A from Hammer and Menzel (1995); Figure 1A.

Multiple neurons are found in the subesophageal ganglion (SOG) that respond to sucrose stimulations at the tip of the proboscis. A group of 10 second-order neurons belong to a category of neurons with unique anatomical features (Schroter et al., 2007). The somata of these neurons lie in the ventral midline of the ganglion and branch symmetrically on both sides of the ganglion. They are called ventral unpaired median neurons (VUM neurons). The sucrose response differs from the brisk discharge of all the other neurons found in the SOG by a sloppy, long-lasting excitation. The VUM neurons resemble both anatomical and functional properties of unpaired neurons in the ganglia of insects that have their somata in the dorsal midline [dorsal unpaired median neurons (DUM) neurons] (Pflüger, 1999). The DUM neurons modulate sensory processing, expression of motor patterns, and arousal states. Six of the 10 VUM neurons of the honeybee SOG send their symmetrical dendrites into both sides of the brain with arborizations in the ALs, the lip regions of the MB and the lateral horns (Figure 3). One neuron, the VUMmx1 (neuron 1 with its soma in the maxillary neuromer of the SOG) was selected for further intracellular recordings and stimulation experiments. The hypothesis was tested whether VUMmx1 servers the function of reward in olfactory conditioning (Hammer, 1993). Intracellular depolarization leading to spike activity replaces the sucrose US. The backward pairing, in which intracellular depolarization preceded the odor stimulus failed to induce odor learning. Most interestingly, VUMmx1 activity in normal odor conditioning leads to an increase of response to the odor. So, the reward neuron itself displays pairing specific enhancement of CS response. Furthermore, the appearance of a predicted US as consequence of former learning, reduces the response to the US. Thus, VUMmx1 changes its response to the learned odor and codes the prediction error rather similarly to dopamine neurons in the mammalian ventral tegmentum (Schultz and Romo, 1990). It was concluded that VUMmx1 represents the reward function in associative odor learning in honeybees (Hammer and Menzel, 1995). Interestingly, the evaluating pathway in *Drosophila* is related to dopaminergic neurons (DANs) that provide input to the MB lobes (Aso and Rubin, 2016) and serve both appetitive and aversive learning (Perisse et al., 2013). The DANs are also known in the bee brain reaching predominantly the peduncle (Mercer and Flanagan, 1988; Blenau and Erber, 1998), and they appear to mediate aversive but not appetitive learning (Vergoz et al., 2007). However, these observations do not exclude a role of DANs in appetitive learning also in bees. Octopamine neurons in the vicinity of the MB lobes were found to drive a subset of DANs that are involved in appetitive learning (Burke et al., 2012). VUM neurons projecting to the input side of the MB in *Drosophila* were described (Busch et al., 2009) but it is not known yet whether they are involved in appetitive learning. Thus, the different regions of the MB, different neurons, and different transmitters represent the evaluating circuits reaching the MB. As long as we lack information on the role of the VUM neuron in *Drosophila*, we have to assume that the honeybee brain contains an additional circuitry for reward learning.

### Anatomical and Functional Converging Sites

The CS and US pathway converge at three sites in the brain of the bee, the AL, the lip region of the MB and the later protocerebrum (LP) (Figure 3B). There might well be other converging sites, e.g., in the SOG, since the VUMmx1 branches there too, and potentially contacts downstream higher order olfactory neurons that reach the SOG either directly from the AL, or *via* the MB extrinsic neurons (ENs). In addition, there could be second-order reward neurons that could converge with higher order olfactory neurons. Multiple convergence sites lead most likely to multiple memory trances. We shall focus our search for the memory trace on two of the convergence sites, the AL, and the MB.

Odors are coded in the bee brain by hierarchically organized neural activities in multiple neurons that encode the identity of the odor in a combinatorial way (Galizia and Menzel, 2000; Yamagata et al., 2009). The related memory traces are, therefore, expected to reflect these properties in spatially distributed patterns of change in synaptic strength. We searched for these patterns of change and focused on the AL and the MB lip because they were accessible to our Ca imaging methods.

## Memory Traces

### The Memory Trace in the Antennal Lobe

The strength of the excitation in AL glomeruli activated by the CS+ increases and decreases for the CS– (Faber et al., 1999). All glomeruli involved in coding the CS+ or CS– appear to be involved equally indicating that the memory trace at the level of the first-order synaptic contacts are changed in an associative way. These changes decorrelate the odor code for learned odors and make them less similar. A comparison of the combinatorial patterns of odor codes in the AL and behavioral discrimination scores established a close quantitative link (Deisig et al., 2010). Furthermore, the associative learning effect is generalized to a control odor in both imaging experiments and in behavior.

The AL glomeruli contain thousands of synapses (Shepherd and Grillner, 2018, chapter 28), and it is unknown which of these synapses are involved in the memory trace. The AL ENs such as the m- and l-ALT read out these associative changes and change their response patterns accordingly (Denker et al., 2010). The extracellular multi-unit recordings combined with local field potential analyses document spike rate increases and decreases in responses to CS+, CS–, and a control odor. The ensemble activity changes most strongly for CS+. Furthermore, the local field potential power increases for CS+ in the 15–40-Hz frequency band, and decreases for frequencies above 45 Hz. This learning related power change correlates with the size of the neuronal ensemble phase-locked to the particular frequency. Thus, less units were entrained to the higher frequency band, and more units to the lower band after learning. Although these results do not inform us about the synaptic location of associative plasticity in AL, they document a restructuring of the odor coding network in the AL.

One might argue that such a memory trace may drastically change the olfactory world uncoupling the bee from reliable and constant sensation. However, neurons leaving the AL project not only to the MB but also to the LH (Schmuker et al., 2011; Zwaka et al., 2016). Since the MB lies in a parallel pathway of odor processing (Figure 3B), and only the neurons projecting also to the MB (l-ALT and m-ALT) may undergo associative plasticity in the AL, the bee will also receive information independent of odor experience. Support for this interpretation comes from the finding that pheromones are stably coded (Sandoz et al., 2007). In addition, it has been argued that the associative changes in the AL may predominantly play a role in improving olfactory coding rather than driving olfactory behavior (or possibly only transiently) (Galizia, 2014). This notion is relevant in the context of what we consider to be part of the memory trace. Are changes of the coding scheme of sensory stimuli not an essential component of sensory coding leading to improved discrimination, change of generalization profiles and transfer to new categories as in rule learning?

### Memory Traces in the MB

**Figure 4:**
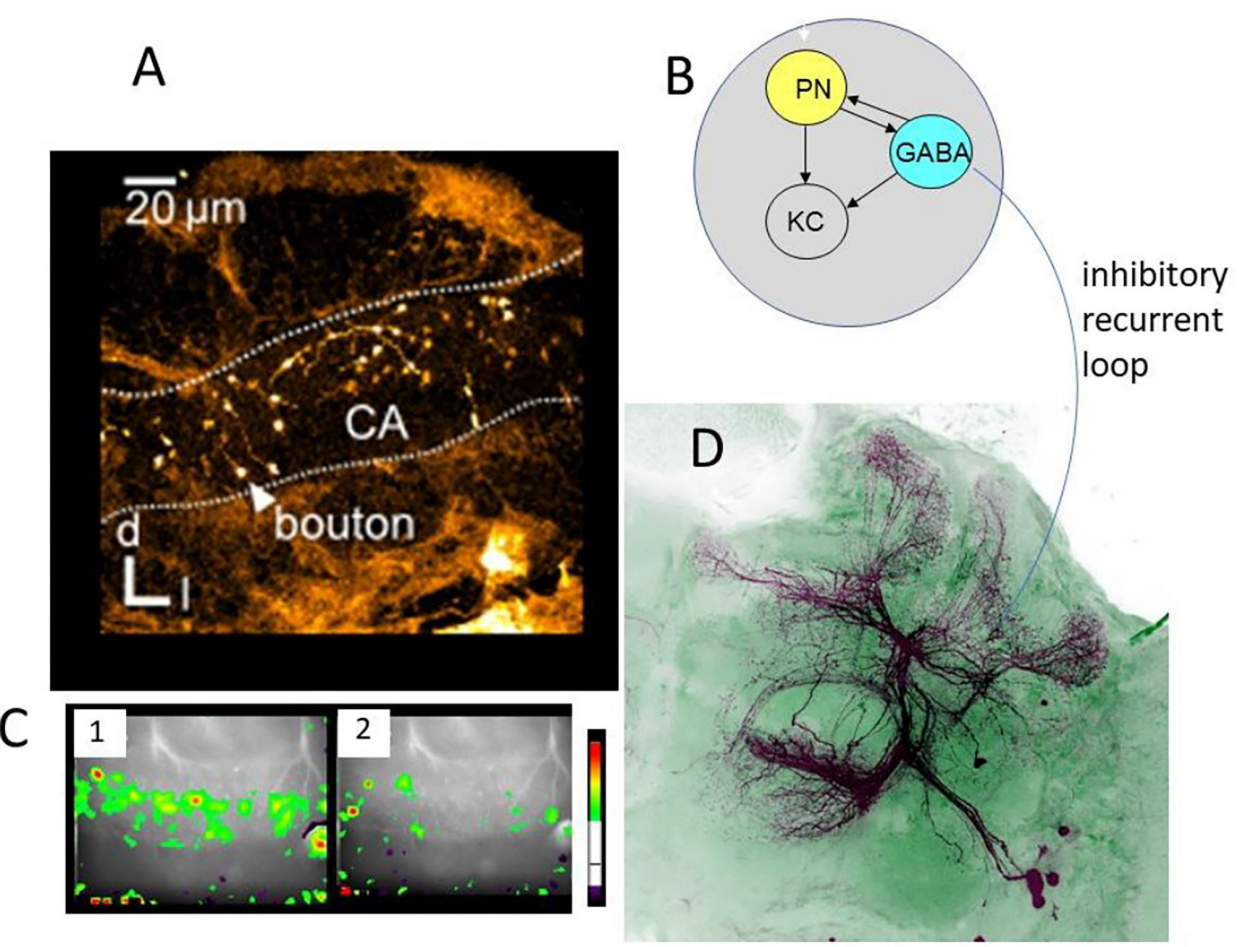
Synaptic organization of boutons in the MB lip. **(A)** Multiple boutons of one neuron in the lateral antenno-lobula tract l-(ALT). CA, calycal lip region; d, dorsal; l, lateral (Haenicke et al., 2018, Figure 2D). **(B)** The scheme of the synapses related to the bouton. The gray area indicates the area served by en-.passant synapses of the VUMmx1. PN, presynaptic site of the projection neuron; KC, postsynaptic spine of Kenyon cells; GABA, GABA immunoreactive sites of the PCT neuron. **(C)** The pattern of Ca activity upon odor stimulation. The activation patterns of two different odors are shown (Haenicke et al., 2018, Figure 2E). **(D)** PCT neurons. The somata lie in the lateral part of the brain. They receive their input in the output regions of the MB, the lobes, and feed–back to the lip region of the MB.

**Figure 5:**
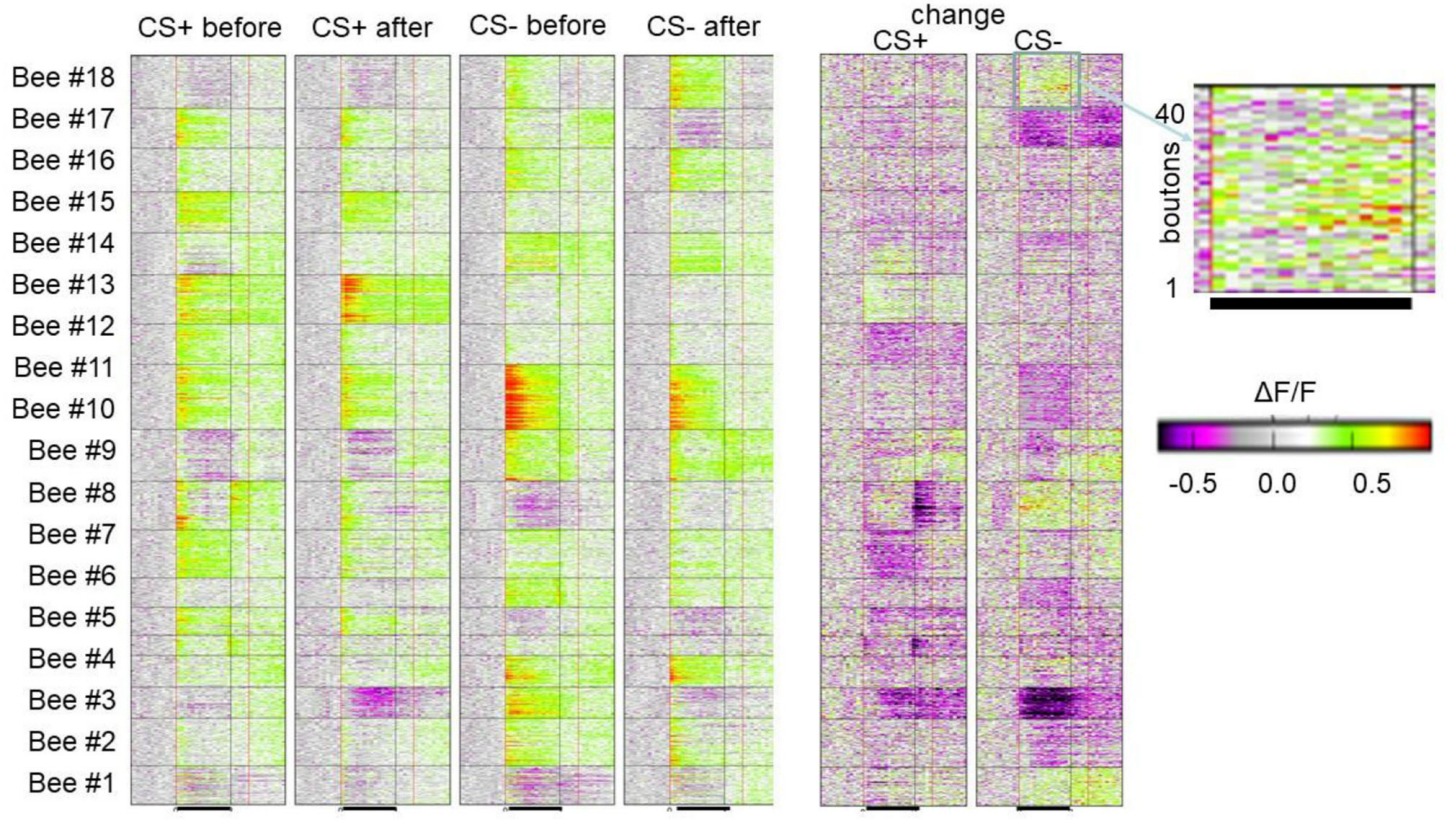
Associative plasticity of boutons in the lip of the MB. Each line in each of the 18 horizontal boxes gives the time course of Ca activity before, during and after odor stimulation of 18 animals. The blown-up picture on the right side shows the bouton wise color-coded change of response. Forty boutons are displayed. The black line marks the odor stimulation. As indicated, the four columns give the color-coded Ca activity of the 18 animals before and after conditioning separately for the CS+ and SC–. The two columns show the change of Ca response in false colors. The bar indicates the color code applied to both 4 columns to the left and two columns to the right (Haenicke et al., 2018; see Figures 3, 4).

**Figure 6:**
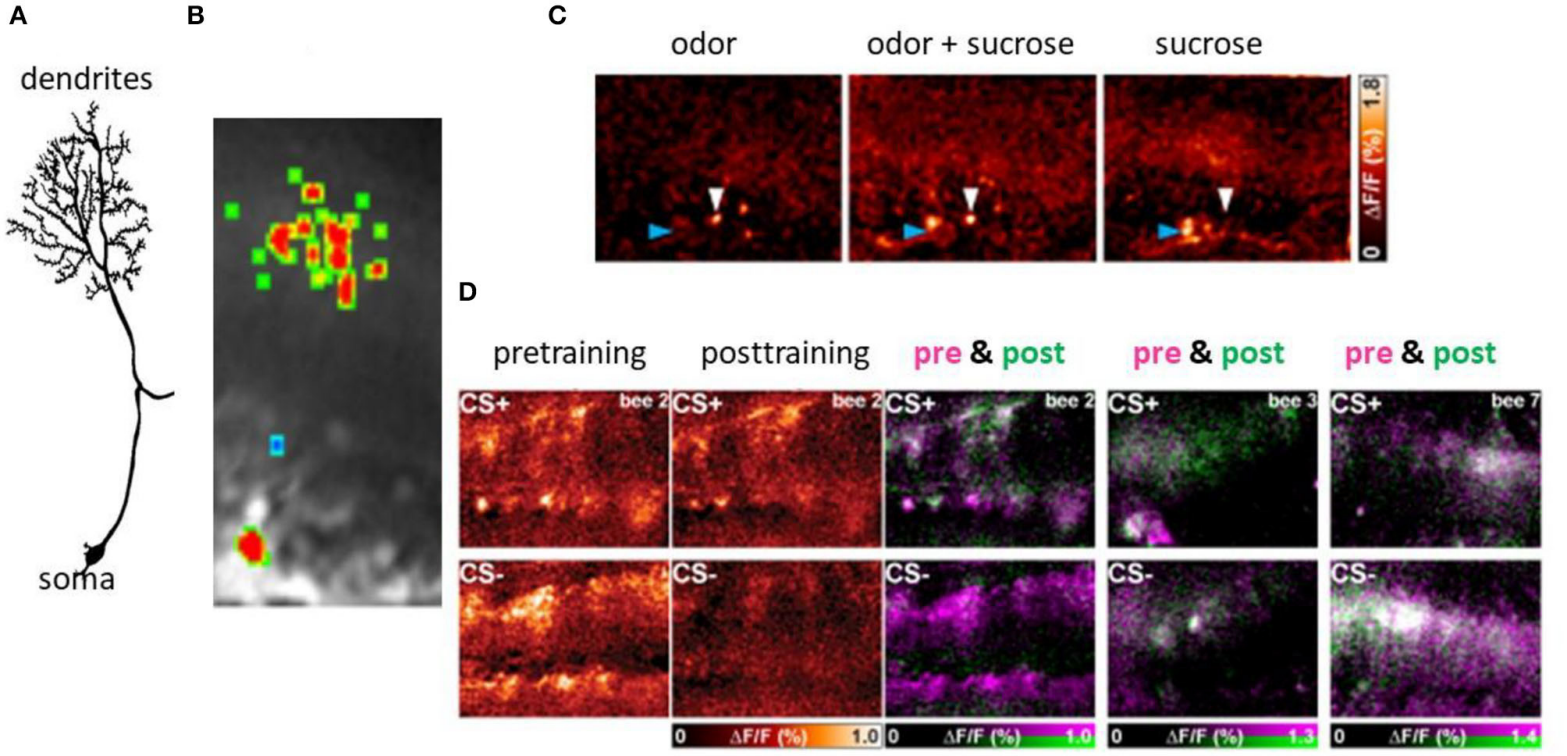
Associative plasticity at the postsynaptic side of the KCs in the lip region of the MB. **(A)** A KC showing its spiny dendrites, the neurite and the soma. **(B)** The Ca imaging of the activity in the spines of a KC. **(C)** Sparse coding of odors in KCs both at the level of spines (blue arrow head) and the somata (white arrow heard). The bar on the right side gives the false color code from dark red (no Ca activity) to white (high Ca activity). **(D)** Changes of Ca activity during differential conditioning. The somata appear in the lower part of each picture, that of the dendrites in the upper part. The most left pair of figures shows the false color-coded Ca activity before conditioning for both CS+ and CS–, and the next pair the Ca activity after conditioning for the CS+ and the CS–. The Ca activity is expressed in the normalized change of Ca activity (Δ*F*/*F* ranging from zero to 1.0). The next three pictures show the learning effect in three animals separately for CS+ (upper pictures) and CS– (lower pictures). The Ca activity before conditioning is color-coded in pink and that after conditioning in green. The Ca activity only before conditioning will appear in pink indicating loss of activity, Ca activity only after conditioning will appear in green indicating a gain of activity. The Ca activity during both pre- and post-training phase will appear in white (no change) (Szyszka et al., 2008, see Figure 4A).

Other than the AL, the lip region of the MB allows for a finer grain analysis of the synaptic changes during memory formation (Figures 4–6). Here, the olfactory PNs form rather large presynaptic boutons at postsynaptic spines of MB intrinsic neurons, the KCs. The boutons receive input from inhibitory recurrent neurons, the protocerebral calycal tract (PCT) neurons (Figures 4B,D). Boutons are also presynaptic to PCT neurons forming a synapse specific local feed–forward, feed–back system (Figure 4B) (Ganeshina and Menzel, 2001; Zwaka et al., 2018). The whole complex of each bouton is served by en-passant presynaptic endings of the VUM neuron (Schröter and Menzel, 2003; Sinakevitch et al., 2013). The stimulating the bee with odors leads to odor specific patterns both at the presynaptic bouton sites (Figures 4, 5) and the postsynaptic KC site. These anatomical and functional units of boutons are likely to serve as an essential element of the memory trace because they are the convergence sites of the CS and US pathways, and they receive input from high order interneurons that read out the memory traces of the MB. Ca-imaging of the pre and the postsynaptic sites allows to address the question of how the semantics of the olfactory memory trace may work in one of the several memory traces.

### Presynaptic Site

As in the experiments with Ca imaging of the AL we applied a differential training scheme pairing the CS+ odor forward with sucrose US and the CS– odor specifically not pairing with US. We monitored the PER to both odors and to the US. In behavior, a high positive correlation was found between the frequency of PER for CS+ and a negative correlation with CS– (Haenicke et al., 2018). Figure 5 shows the results of all boutons imaged in 18 animals for the CS+ and CS– before conditioning and after conditioning together with the change for the CS+ and CS– in false colors. The area covered during Ca imaging comprised approximately a 40th part of the lip region in the frontal aspect of the MB. As expected, most of the boutons respond with excitation to the odors, but some boutons (e.g., in animals 1, 2, 3, 8, 9, 14, and 18) respond with inhibition. These inhibitory responses result most likely from the inhibitory input *via* the PCT neurons, and since PCT neurons were shown to change their responses in the course of olfactory learning (Haehnel and Menzel, 2010; Filla and Menzel, 2015) they carry information about former odor experience possibly notifying already learned odors to the MB input *via* inhibition (see below). A majority of boutons change their responses only in one direction, i.e., either increase or decrease their responses during conditioning, and a small part of boutons change in both directions. The response reduction appears in about a third of the boutons, suggesting a dominant learning effect through some inhibitory mechanism. The probability of each type of change is roughly equal for CS+ and CS– odors, suggesting no apparent contrasts between rewarded and unrewarded odors in this regard. In an additional step, we quantified neural plasticity *by ΔNR* that captures the absolute changes of bouton activity. In the same way, we quantified the changes in behavior (Δ*CR*) of each individual bee during and after training and found a positive correlation between learning induced neural plasticity of CS+ odor and behavioral performance. In contrast, the CS– plasticity did not show a significant correlation with the behavior. The correlation between the absolute amount of plasticity with the behavioral change highlights the importance of both up- and down-regulation of bouton activities in learning. The up- and down regulation was also found in AL ENs (Denker et al., 2010) leading to little or no change across multiple neurons. This form of stability appears as a basic principle of associative plasticity (see e.g., Fernandez et al., 2009; Haehnel and Menzel, 2010; Rath et al., 2011; Strube-Bloss et al., 2011, 2016; Filla and Menzel, 2015). The neural plasticity without altering overall excitation may therefore be a general mechanism seen in the insect brain, presumably to achieve effective memory encoding in the limited coding space with less energy consumption.

### Post Synaptic Sites in the MB

Each of the paired MBs contains ~300,000 neurons, the KCs. We focused on the clawed KC whose dendrites branch in the lip region. Odors are sparsely coded in KCs, both at the level of the somata and the dendrites (Szyszka et al., 2005). On average around 10% of the neurons respond to a given odor, and the pattern is stimulus specific. The time course is phasic leading to only a few spikes in KCs, a property well-known from other insect species (Perez-Orive et al., 2002). Sparse coding in KCs is mediated *via* a GABA_A_ and GABA_B_ Receptor mechanism most likely driven by the GABA immunoreactive PCT neurons (Froese et al., 2014). From a theoretical point of view, the temporal and population sparseness makes KCs potentially well-suited as a memory store since the organization of its input site can be conceptualized as an associative matrix formally comparable to the network of the hippocampus or cortex (Rolls, 2007).

Ca^2+^ imaging of the KC spines in the lip region of the calyx allows us to elucidate learning-related plasticity of this matrix-like circuit (Szyszka et al., 2008) (Figure 6). The stimulus repetition leads to depressed responses in KCs, a form of non-associative plasticity that is counteracted for the CS+ but not for the CS– in differential conditioning. This finding suggests that the meaningless repetitions of stimuli leads to depression, and meaningful (as indicated by the activation of the reward pathway) stimuli compensate depression possibly by selectively facilitating neural responses. Furthermore, KCs are either specifically recruited or eliminated from responding during odor learning leading to a change of odor induced activity pattern in KCs. The gain of activity (recruitment) was observed more frequently for the CS+ and loss of function (elimination) more frequently for the CS-, but both changes occur for both stimuli. The unchanged activity in the course of odor learning was rather rare indicating that learning leads to a drastic rearrangement of odor representation in the MB input. Taken together it is conceivable that the olfactory engram in the MB lip comprises a combinatorial pattern of predominantly enhanced synaptic transmission to KCs but also reduced transmission leading to the conclusion that KCs store a memory trace in stimulus-specific sparse activation patterns.

### Retrieval of the Memory Trace in the MB

The large number of densely packed KCs in the MB converge on a rather small number (a few hundreds) of MB ENs in the three output regions of the MB, the peduncle and the alpha and beta lobes. Rybak and Menzel (1993) characterized eight different groups of ENs. Most of these groups contain around 70. The multiplicity of connections established by these ENs makes it likely that each group serves a different functional retrieval category (Menzel, 2014). This view is supported by the finding that ENs predict the behavioral performance of the individual animal during memory retrieval following differential odor conditioning (Strube-Bloss et al., 2021).

Since many of these ENs receive input across the modality specific regions of the MB it is not surprising that they respond to a large range of sensory stimuli (Homberg and Erber, 1979) indicating a different coding scheme than the highly specific combinatorial sensory code at the input of the MB. For example, one large EN, the peduncle EN #1 (PE1), offers the unique possibility to repeatedly record intracellularly from the same identified neuron during olfactory PER conditioning. The PE1 reduces its responses to the learned odor (Mauelshagen, 1993; Okada et al., 2007). It receives excitatory input across the whole MB reflected by its multimodal sensitivity (Mauelshagen, 1993; Iwama and Shibuya, 1998; Rybak and Menzel, 1998). It also receives inhibitory input from the PCT neurons (Okada et al., 2007). The PE1 neuron develops two categorically different forms of associative plasticity during context-dependent learning tasks. The contexts used were light of different colors or temperature changes, either alone or in combination. We found that bees learn context rules quickly and use them for better discrimination. They also solve a transwitching and a cue/context reversal task (Hussaini and Menzel, 2013). The PE1 responds to the learned olfactory cue with a reduction of spike activity, and the learned context stimuli lead to a response enhancement. These results indicate that PE1 encodes cues and contexts differently and may retrieve different memory traces selectively. The PE1 neuron displays anatomical features close to MB ENs GABAergic ENs in Drosophila (Aso et al., 2014a, see their Figures 15E, 17), but PE1 does not have projections to the other brain hemisphere and it is not GABAergic.

The PCT neurons mentioned above as a recurrent inhibitory loop from the output to the input of the MB integrate the sum of the values associated with both visual context and odor cue (Filla and Menzel, 2015). The presence of the learned context enhances neural responses to the rewarded odor, whereas unrewarded odor responses were further reduced during the unrewarded context. In addition to stimulus valuation, PCT neurons generate a neural error signal after an incorrect behavioral response. Furthermore, PCT neurons show a similar generalization profile in their learned responses to behavioral generalization (Haehnel and Menzel, 2012). This might act as a learning signal in the feedback loop to the MB input. All of these effects were exclusively found in trials in which the animal prepared for a motor response that happened during attentional stimulus selection. Based on these studies, we concluded that PCT neurons constitute a multisensory value integration read-out specifically designed for overt action selection, comparable to neurons in the mammalian prefrontal cortex (Kaping et al., 2011). One could thus speculate that the successful coupling of context/cue and action could be stored in these neurons, and incoming stimuli could directly be evaluated concerning value and appropriate action.

### Lateral Horn

The LH receives input from the AL directly and indirectly *via* the MB. For example, the PE1 neuron projects to the LH and may inform the LH circuit whether an odor had been learned or is novel. The mechanism behind this information was tentatively related to the reduced responses of the PE1 to learned odors, and since the PE1 receives input from GABA immunoreactive PCT neurons (Okada et al., 2007), the reduced inhibition would promote the increase of excitation from both AL and MB ENs for learned odors (Menzel, 2014). This information is highly specific, because the learned response changes of PE1 are categorically different for a learned odor cue and a visual context (Hussaini and Menzel, 2013). Furthermore, it has been argued above that the LH may serve a direct olfactory-motor pathway possibly closely related to innate responses. However, both PNs and the input from the MB ENs are known to change their response properties during olfactory learning. Local cooling experiments shortly after a single olfactory learning trial directed to the lateral protocerebrum did not lead to any impairment of olfactory memory (Erber et al., 1980), but this could either reflect a limitation of the method used to induce retrograde amnesia or an indication that the LH develops associative plasticity at later stages of learning. It was not possible yet to apply Ca imaging experiments to the LA as to the AL and MB in the context of learning experiments. Thus, the interpretation that the LH may not be part of the memory trace but rather a read-out or pre-motor circuit is currently tentative. Galizia (2014) views the existing data from the perspective of a distinction between odor identification (a property of the MB) and of odor evaluation (a property of the LH) both of which are highly experience dependent. This view emphasizes the open question how much and in which sense retrieval pathways are part of the memory trace.

## Concluding Remarks

The physical equivalents of a memory trace is a pattern of synaptic change at the level of synapses. This pattern contains information for neurons that can read it. Components of the stored information can be captured by the human observer if the pattern before learning and that after learning can be compared with respect to what these patterns encode. So far, such an extrinsic decoding procedure is not possible even in an insect brain. In the case of the honeybee MB a rough estimate leads to ~4,000 gained KCs with a total of ~40,000 synapses and roughly similar numbers for lost and unchanged KCs and their synapses in coding an odor memory trace. Future methods may allow us to activate and inactivate specifically the involved synapses allowing to test whether indeed these synapses store the content of the memory, how robust the store is, whether it contains features known from matrix like memories (e.g., graceful degradation, completion, Rolls, 2007). Such an approach may well be in range in work with *Drosophila*. The atlas of its brain has reached the subcellular level (Zheng et al., 2018), and the tools exist to specifically activate subpopulations of neurons, conditions that will be gained for the bee brain only in a further future.

Meanwhile we are only able to compare the small sets of recorded changes in synaptic activity patterns and to evaluate them in multiple ways addressing questions, e.g., do the pattern become less similar with learning, do they correlate with the generalization patterns as seen in behavior, do the synaptic patterns consolidate during sleep (a behavior phenomenon also shown in honeybees (Zwaka et al., 2015). In such a case, a closer link can be established between the levels of extrinsic “memory reading.”

However, any extrinsic reading is highly superficial because of three obvious reasons. First, even if we could read the total pattern of change at a particular site of the brain, we do not know what the other sites contribute to the behaviorally relevant memory. It was, therefore, my motivation to emphasize the multitude of memory traces for even a rather simple task, associative learning of one or two odors at different levels of odor coding. Second, memory retrieval is a highly dynamic process involving state-dependent and modulatory circuits. It will be close to impossible to relate these effects to just the activity pattern during a retrieval test. Context dependent learning retrieval is such a case. The neurons involved in such a retrieval process obviously solve the task by separating categorically between the learned cue and the learned context, but an extrinsic memory reader focusing on one of many sites of the memory trace will not be able to solve this separation. Third and most importantly, the neurons involved in retrieving the memory, e.g., the olfactory PNs or the MB ENs and their joint actions at the level of the LH, are involved in the learning process themselves, and thus in the memory trace. For example, I speculated about a memory trace in PCT neurons and suggested that these neurons may be involved in separating between already learned stimuli (odor cues, visual context) and novel stimuli. This speculation was based on the anatomy of PCT neurons (recurrent loop), their inhibitory transmitter (GABA) and the observation that they coded cue and context categorically differently. A similar speculation was applied to the role of the PE1 neurons at the level of the LH. New methods will make it possible in the future (as is already possible in the fruit fly) to activate or block the function of these neurons or single members of them. If the methods will be developed such that synapses can be addressed specifically and the read-out leads to specific forms of behavior, we shall be informed of whether a hypothesis is supported or not. For example, a neuron in the *Drosophila* brain, the APL neuron, with close anatomical and functional features to the PCT neurons in the bee brain, was manipulated in such a way and effects along these arguments were found but so far on the level of the selected neuron and not the patterns of synapses involved (Liu and Davis, 2009; Eichler et al., 2017; Saumweber et al., 2018).

Ultimately, we need to unravel the semantics of the memory trace and relate it to neural properties. The ideal approach would be to search for these correlates not at the level of sensory coding, pre-motor commands, evaluating and modulatory circuits, but rather at the level of derived components of sensory cues that do not exist prior to any learning. Such derived cues appear during generalization, categorization and rule learning. In such a situation the animal bases its choice on a rule that transcends the stimuli involved in training it. The memory content is thus fully novel and should be accessible in tests with novel stimuli. The strength and specificity of data will be particularly high if the rules run across sensory modalities or are fully abstract as in the case of counting, a cognitive faculty that has not yet been documented for insects. Useful paradigms are listed as follows: Matching-to-sample (or non-matching-to-sample) tests (Zhang et al., 2004; Giurfa, 2019), learning of mirror symmetry from multiple examples (Giurfa et al., 2001; Stach and Giurfa, 2001), configuration of visual stimuli (Giurfa et al., 2003; Stach et al., 2004; Stach and Giurfa, 2005), and learning of trends in reward (Gil et al., 2007) and sequences learning (Zhang et al., 2005; Menzel, 2009). These and other behavioral phenomena are well-documented for the honeybee but are lacking for *Drosophila*.

Does a memory exist if it is not retrieved? If the knowledge stored in memory does not guide behavior, a behavioral biologist cannot know whether memory exists (and may thus define memory by its retrievability). However, a neuroscientist cannot help but assume that the knowledge stored in memory continues to exist during time periods when it is not retrieved, because the physiological determinants of memory are thought to be independent of whether the animal performs the corresponding behavior. Two concepts are particularly relevant in this context, memory consolidation and shift of memory to other brain areas. Memory consolidation during sleep is accompanied by the activation of circuits that had been involved in learning (e.g., place cells in the mammalian hippocampus and visual cortex (Ji and Wilson, 2007). Reminder stimuli possibly involved in activation the memory trace during sleep lead to enhanced memory consolidation in vertebrates including man (Diekelmann and Born, 2010) and honeybees (Zwaka et al., 2015). The sleep conditions (e.g., slow wave sleep in humans) have the capacity to activate selectively recently acquired memory traces. Thus, there must be “something” physical that exists for being activated. The consolidation moves the memory traces to other circuits, a phenomenon well-documented for vertebrates (Battaglia et al., 2011) and the bee (Sandoz et al., 2002). The transfer between brain areas alters the memory and stabilizes it. The physical substrate for the brain intrinsic processes must be accessible independent of a behaviorally relevant retrieval process. Irrespective of the unresolved questions in separating memory formation and memory retrieval processes, the body of evidence is overwhelming, proving that neural traces are indeed induced by the learning process independent of retrieval, and consolidation has a physical basis in the structuring and restructuring processes of neural net properties.

The many facets of memory are reflected in the many terms used to capture them. Are there 256 different kinds of memory, as Tulving (1972) asked? Irrespective of whether we divide up memories according to time, cellular mechanisms, brain structures involved, categories of contents, type of learning, or type of retrieval, we always imply that memory directs behavior *via* the process of retrieving information. Thus, the semantics of memory trace provides the knowledge base for behavioral guidance by the retrieval process. One question that needs ultimately answered is as follows: How do we go about measuring the knowledge stored in memory? We simply do not know (yet?).

## Author Contributions

The author confirms being the sole contributor of this work and has approved it for publication.

## Conflict of Interest

The author declares that the research was conducted in the absence of any commercial or financial relationships that could be construed as a potential conflict of interest.

## Publisher's Note

All claims expressed in this article are solely those of the authors and do not necessarily represent those of their affiliated organizations, or those of the publisher, the editors and the reviewers. Any product that may be evaluated in this article, or claim that may be made by its manufacturer, is not guaranteed or endorsed by the publisher.
